# Safety of Early Discharge in Patients Undergoing Urgent Transcatheter Aortic Valve Implantation

**DOI:** 10.3390/jcm15187215

**Published:** 2026-09-17

**Authors:** Paraskevi Trivilou, Omran Abukhalaf, Moad El-Haddad, Muntaser Omari, Debbie Stewart, Sarah Lamb, Timothy Cartlidge, Mohamed Farag, Rajiv Das, Richard Edwards, Azfar Zaman, Mohammad Alkhalil

**Affiliations:** 1 Cardiothoracic Centre, Department of Cardiothoracic Services, Freeman Hospital, Freeman Road, Newcastle-Upon-Tyne NE7 7DN, UKomran.abukhalaf@nhs.net (O.A.); richard.edwards21@nhs.net (R.E.);; 2 Translational and Clinical Research Institute, Newcastle University, Newcastle-Upon-Tyne NE7 7DN, UK

**Keywords:** TAVI, aortic stenosis, in-hospital, mortality, vascular complication, stroke, pacemaker

## Abstract

**Background:** Patients undergoing urgent transcatheter aortic valve implantation (TAVI) represent a clinically high-risk population with increased peri-procedural morbidity. Whether an early discharge strategy is feasible and safe remains unclear. We evaluated the feasibility of early discharge in patients undergoing urgent transcatheter aortic valve implantation. **Methods:** This single-centre observational cohort study included 279 consecutive patients undergoing urgent TAVI (mean age 81 ± 7 years; 155 [56%] male). Patients were stratified according to post-TAVI length of stay into next-day discharge or prolonged hospitalisation (>1 day). Data were prospectively collected and the primary end point was 30-day all-cause mortality. **Results:** Among 279 urgent TAVI patients, 62 (22%) were discharged the next day and 217 (78%) had prolonged hospitalisation. Baseline clinical and echocardiographic characteristics were similar between the two groups. Among the 279 patients surviving to discharge assessment, 30-day mortality was 0.4%, with no significant difference between the next-day and longer-stay groups. Patients in the short hospital stay group had an 11% complication rate compared to 30% in the long stay one. Unlike stroke or pacemaker implantation, vascular complication was an independent predictor of extended in-hospital length of stay [Beta coefficient 3.41, 95% CI (0.98 to 5.85), *p* = 0.006]. **Conclusions:** Next-day discharge appears feasible in carefully selected patients undergoing urgent TAVI and was not associated with excess 30-day mortality in this observational cohort. Prospective multi-centre studies are required to validate these findings and establish standardised discharge criteria.

## 1. Introduction

The numbers of patients undergoing TAVI continues to increase worldwide. As operator experience and results have improved, the proportion of patients with acute decompensation from severe aortic stenosis requiring urgent TAVI has also increased [1,2]. Severe aortic stenosis (AS) requiring in-hospital admission is associated with worse prognosis compared to elective planned transcatheter aortic valve implantation (TAVI) [3,4,5]. Therefore, patients undergoing urgent TAVI represent a clinically distinct and higher-risk population.

Contemporary guidelines recommend aortic valve intervention in patients with symptomatic severe aortic stenosis, with the choice between transcatheter and surgical aortic valve replacement individualised through multidisciplinary Heart Team assessment. Patient selection should incorporate age and estimated life expectancy, surgical risk, comorbidity and frailty, anatomical suitability for transfemoral TAVI, procedural feasibility, and patient preference. In this context, patients who present with acute clinical deterioration requiring continued inpatient management and valve intervention during the index admission represent a distinct population from those undergoing planned elective TAVI [1].

Urgent or emergent TAVI is associated with greater comorbidity burden and higher rates of early and late mortality compared with elective procedures. Data from the STS/ACC TVT registry highlighted a 20–30% increased risk of 30-day as well as one-year mortality [3]. Moreover, urgent TAVI was associated with higher rates of acute kidney injury and other peri-procedural complications [3,4,5]. Similarly, numerous studies have highlighted that patients undergoing TAVI during admission for acute decompensated heart failure experience longer hospitalisation, larger healthcare costs, and resource utilisation [6].

Notably, contemporary strategies aimed at reducing in-hospital length of stay have focused on carefully selected elective TAVI patients. Same-day and next-day discharge protocols have been shown to be safe in low-risk, clinically stable patients undergoing uncomplicated procedures, contributing to improved hospital efficiency without compromising short-term outcomes [7,8,9,10]. However these studies have largely excluded patients undergoing urgent TAVI, given their perceived high procedural risk.

This study aimed to assess clinical and procedural characteristics as well as outcomes for patients undergoing urgent TAVI procedures according to their hospital length of stay post-TAVI procedure.

## 2. Methods

This was a single-centre observational cohort study of consecutive patients with severe symptomatic aortic stenosis admitted to hospital with decompensated heart failure, syncope, or significant symptom burden who subsequently underwent urgent TAVI. For this study, urgent TAVI was defined as TAVI performed during an unplanned hospital admission in a patient with severe symptomatic native aortic stenosis or a degenerated bioprosthetic aortic valve who required continued inpatient management because of acute decompensated heart failure, syncope, or significant symptom deterioration, and in whom discharge before valve intervention was considered clinically inappropriate. Data were collected prospectively in a dedicated institutional database maintained by a specialist TAVI nursing team, and they were regularly audited to ensure accuracy and completeness. Clinical, procedural, and echocardiographic data were systematically recorded and analysed. The study was exempted from institutional board review and the formal ethics process as it was part of a clinical audit. Patients were consented to have their clinical procedures and data can be used anonymously.

Between January 2024 and December 2025, consecutive patients with severe native aortic stenosis or degenerated bioprosthetic aortic valves who were admitted as inpatients and treated with urgent TAVI were included. Patients were referred from five different centres due to acute symptom deterioration requiring inpatient management. All cases were discussed by the specialist TAVI team and deemed suitable for TAVI in accordance with current guideline recommendations. Patients were included irrespective of surgical risk, valve platform, left ventricular function, or haemodynamic status at presentation.

Pre-procedural planning included multidetector computed tomography in all patients, and images were analysed using a dedicated software for annular sizing and vascular access assessment (3mensio Structural Heart, version 10.6, 3mensio Medical Imaging, Maastricht, The Netherlands) [11,12].

Transfemoral-access TAVI procedures were performed by two experienced operators with the choice of valve type, use of pre- or post-dilatation, and peri-procedural management left to operator discretion. Both self-expanding and balloon-expandable valve platforms were used according to anatomical suitability and clinical judgement.

### 2.1. Post-Procedural Discharge Strategy

Post-procedural length of stay was determined by clinical recovery, haemodynamic stability, absence of procedural complications, and treating physician assessment. Patients were stratified into two groups according to discharge timing: (i) next-day discharge, defined as short in-hospital stay, and (ii) long in-hospital stay, if patients were in hospital for at least two consecutive nights.

No formal pre-specified discharge protocol was mandated; however, early discharge was encouraged and considered if the clinical team deemed the patient to have stable rhythm, vascular access haemostasis, good functional recovery and safe mobilisation.

The primary outcome of the discharge-stratified analysis was 30-day all-cause mortality among patients who survived the immediate procedural and in-hospital period and reached the point at which a post-TAVI discharge strategy could be considered. Patients who died during the procedure or before discharge could not be assigned to either the next-day or prolonged-hospitalisation group and were therefore excluded from the discharge-stratified comparison. This analysis should consequently be interpreted as a conditional analysis among patients surviving to discharge rather than an estimate of overall mortality following urgent TAVI. To assess overall early mortality following urgent TAVI, a sensitivity analysis was additionally performed in the complete consecutive urgent-TAVI cohort from the time of the procedure, including procedural and in-hospital deaths. Patients who experienced major in-hospital complications, including stroke, vascular complications, or requirement for permanent pacemaker implantation prior to discharge decision, were included in the analysis and allocated according to their final discharge status. Major complication was defined as the composite of stroke, new pacemaker, and major vascular complication as defined according to the Valve Academic Research Consortium-3 (VARC-3) criteria [13].

No formal pre-specified numerical discharge algorithm was mandated during the study period. However, all patients underwent structured clinical assessment following TAVI, and next-day discharge was considered only when the treating clinical team was satisfied that adequate post-procedural recovery had occurred. The decision remained individualised and was made by the treating team rather than being determined by a prospectively mandated discharge protocol. Clinical domains routinely assessed included:

1. Haemodynamic stability without ongoing intravenous vasopressor or significant intravenous therapy requirements.
2. Stable cardiac rhythm and absence of high-grade atrioventricular block or other conduction abnormality requiring intervention.
3. Satisfactory vascular access site haemostasis without evidence of significant bleeding or vascular complication.
4. Adequate functional recovery including safe mobilisation.
5. Assessment of renal function and haemoglobin when clinically indicated.
6. No ongoing significant oxygen requirement beyond the patient’s baseline.
7. Adequate oral intake and clinical recovery.
8. Assessment of the patient’s home circumstances and ability to return safely to their usual environment including availability of appropriate support where required.

### 2.2. Statistical Analysis

Descriptive statistics and categorical comparisons were performed using chi-square or Fischer exact tests as appropriate. Continuous variables were presented as mean ± standard deviation or median ± interquartile range, as appropriate, and were compared using the unpaired *t*-test or the Wilcoxon rank-sum test.

To investigate the relationship between procedural complications and in-hospital length of stay, univariate and multivariable linear regression analysis were performed. Clinically relevant variables such as age, gender, the Society of Thoracic Surgeons (STS) score, left ventricle (LV) function, aortic valve severity, transcatheter heart valve platform, and procedural complications were entered into the final multivariable analysis. Three models were reconstructed to assess individual procedural complications and their influence on in-hospital length of stay. A *p* value of <0.05 was considered significant. All statistical analyses were performed using SPSS 31 (IBM Corp., Armonk, NY, USA).

## 3. Results

**Figure 1:**
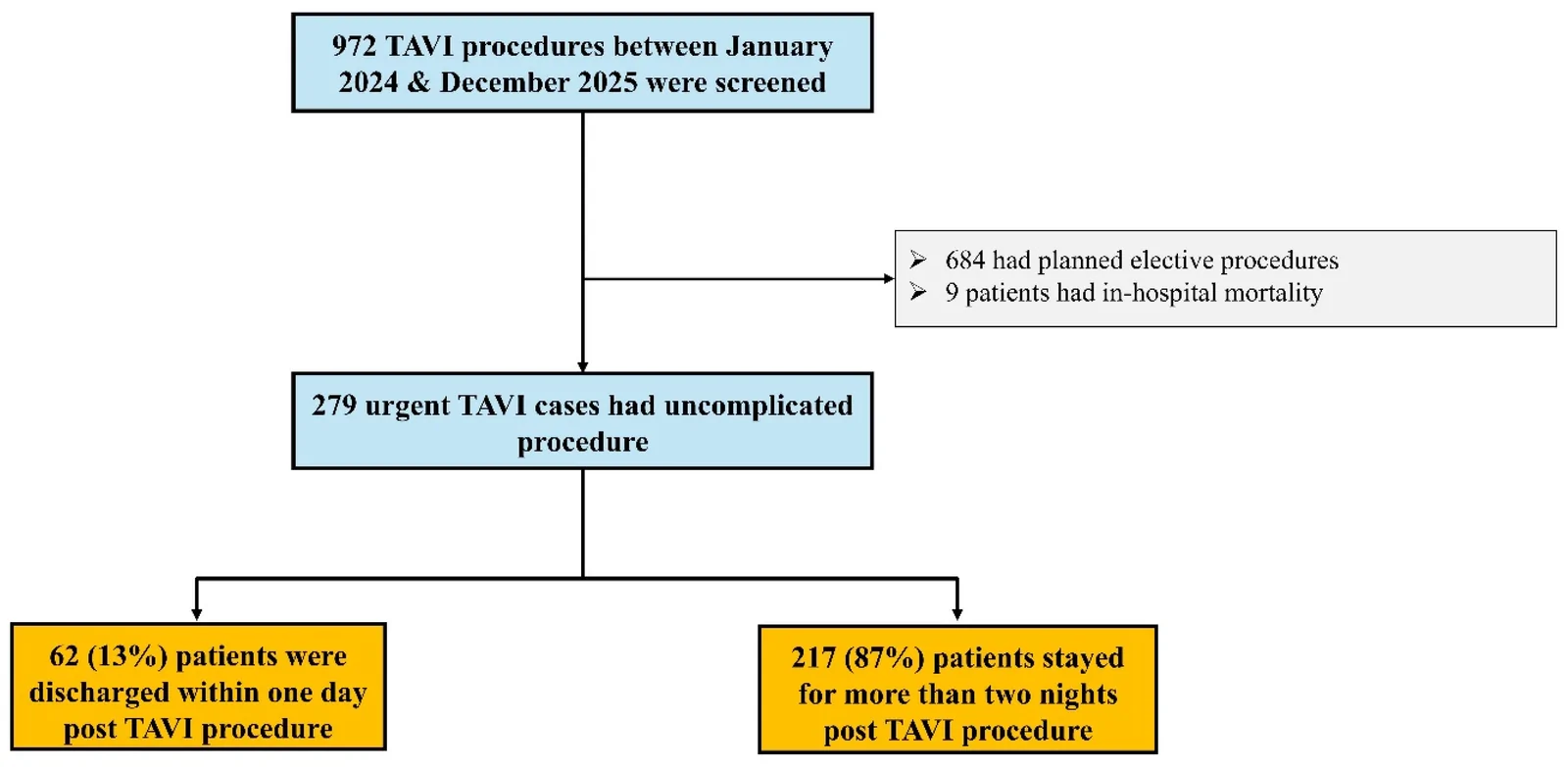
Study flow chart.

The study flow chart is presented in Figure 1. Over the study period, 279 patients underwent in-hospital transfer for an urgent TAVI procedure: of these, 62 (22%) patients were discharged the following day (short in-hospital stay) while 217 (78%) patients stayed for at least two consecutive nights (long in-hospital stay) (Figure 1).

The mean age was 81 ± 7 and 56% were males. Patients in the long in-hospital stay were more likely to be diabetic (36% vs. 24%) or with previous pacemaker (8% vs. 2%), but less frequently to have atrial fibrillation (43% vs. 30%). There was no statistical difference in the prevalence of bundle branch block between the two groups, for LBBB (11% vs. 19%, *p* = 0.17) or for RBBB (11% vs. 15%, *p* = 0.52). Baseline clinical characteristics are presented in Table 1.

There was no difference in LV function or aortic valve severity using mean gradient or aortic valve area between patients in the short versus long in-hospital stay (Table 2). Similarly, echocardiographic measurements were comparable between the groups following TAVI procedures.

**Figure 2:**
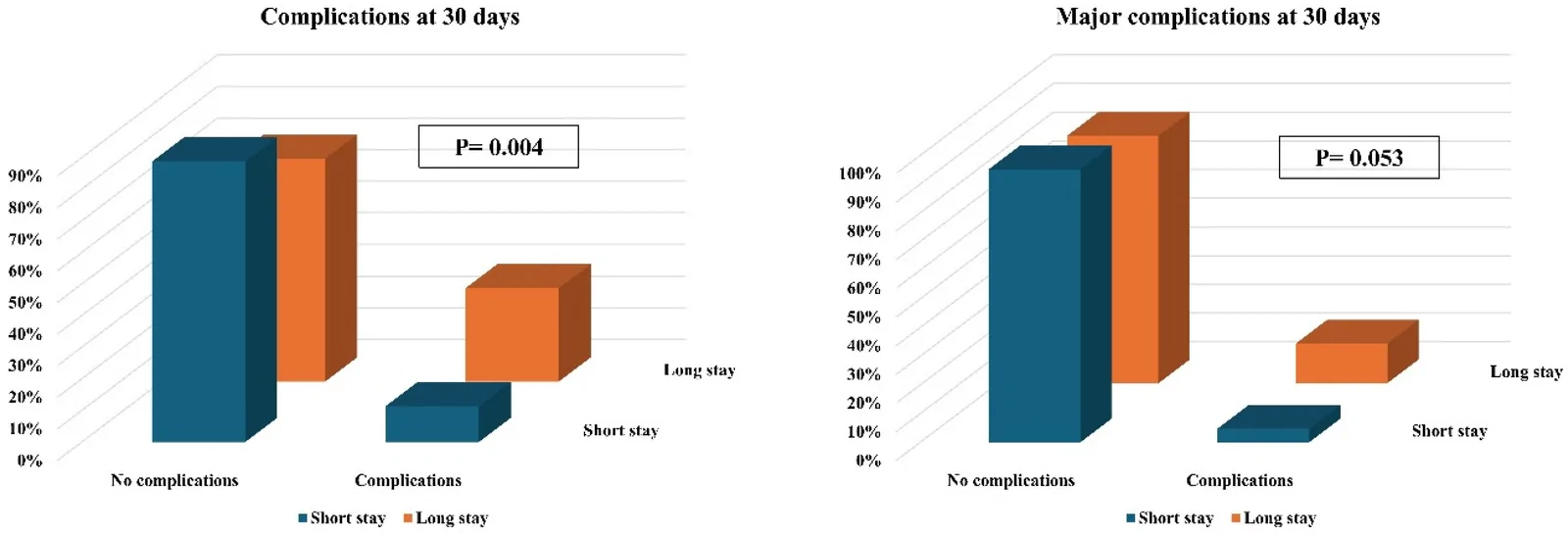
The primary endpoint in patients undergoing urgent transcatheter aortic valve implantation (TAVI). A Kaplan–Meier curve of the cumulative incidence of all-cause death, 30 days follow up.

**Figure 3:**
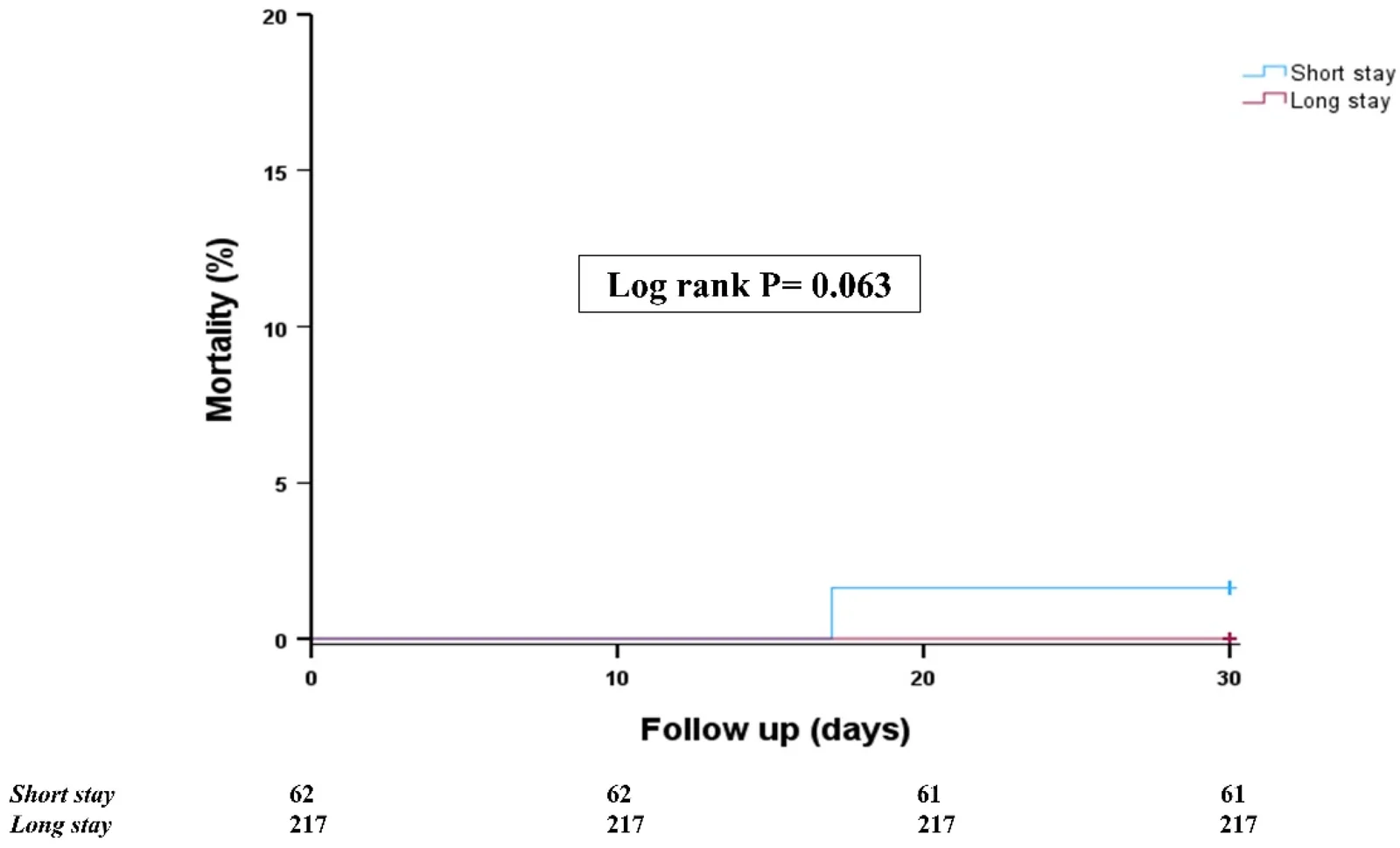
The incidence of any or major complications in the study. Major complication was defined as the composite of stroke, new pacemaker, and major vascular complication as defined according to the Valve Academic Research Consortium-3 (VARC-3) criteria.

Among the 279 patients who survived to discharge assessment and were included in the discharge-stratified analysis, 30-day mortality was 1/279 (0.4%). There was no statistically significant difference in 30-day mortality between patients discharged the following day and those with prolonged hospitalisation (1.6% vs. 0%, *p* = 0.22) (Figure 2). (Figure 2). Patients who were ultimately discharged the following day had a lower observed incidence of any or major complications than those requiring prolonged hospitalisation (11% vs. 30%, *p* = 0.004) and (14% vs. 5%, *p* = 0.053), although the latter did not reach statistical significance (Figure 3). The individual components of major complications were comparable between the two groups (Table 3). This comparison is descriptive, as discharge timing was determined following assessment of post-procedural recovery and complications.

To evaluate whether specific complications contributed to an extended length of stay, we constructed three multivariate linear regression models to assess the relationship between stroke (model 1), vascular (model 2), or pacemaker (model 3) and in-hospital length of stay. In the first model, stroke was not an independent predictor of prolonged in-hospital stay [Beta coefficient 1.57, 95% confidence interval (CI) (−1.65 to 4.79), *p* = 0.34] (Table 4). Similarly, pacemaker was not associated with extended in-hospital stay [Beta coefficient 1.69 95% CI (−0.45 to 3.82), *p* = 0.12]. On the other hand, vascular complication was associated with additional 3.4 in-hospital stay [Beta coefficient 3.41, 95% CI (0.98 to 5.85), *p* = 0.006]. The STS score was also marginally associated with prolonged in-hospital stay [Beta coefficient 0.38, 95% CI (0.12 to 0.64), *p* = 0.004], which was consistent across the three models. Age, gender, LV function and the valve platform were not associated with duration of in-hospital stay.

## 4. Discussion

The main findings of this observational study are: (1) next-day discharge was achieved in approximately one-fifth of patients who survived urgent TAVI to discharge assessment; (2) among these selected patients, next-day discharge was not associated with excess 30-day mortality compared with prolonged hospitalisation; and (3) vascular complications were associated with longer in-hospital stay unlike stroke or pacemaker. Importantly, discharge timing was determined according to post-procedural clinical recovery and was not randomly assigned; therefore, differences between discharge groups should not be interpreted as demonstrating a causal benefit or greater safety of next-day discharge.

Recent studies have illustrated the feasibility of early discharge for patients undergoing planned elective TAVI [7,14,15]. These studies demonstrated the safety of same-day discharge, including for patients with self-expanding platforms [7,16,17]. However, early discharge protocols excluded patients who were transferred for urgent in-hospital TAVI. This group of patients presents with more comorbid conditions, with higher procedural risk that precludes them from early discharge strategies. Our study demonstrates that next-day discharge was feasible in a selected subgroup of a single-centre cohort of patients undergoing urgent TAVI following hospital admission for acute symptom deterioration. Despite their higher-risk clinical profile, patients who were discharged after 24 h of in-hospital stay did not report excess mortality, and had comparable echocardiographic outcomes compared to patients who had extended hospitalisation. These findings challenge the traditional assumption that urgent TAVI necessitates extended post-procedural monitoring and suggest that streamlined discharge pathways may be appropriate even in non-elective settings.

Our data highlights that it is possible to early-discharge patients who were transferred for urgent in-hospital TAVI. This is important as this cohort is considered to be at high risk following TAVI procedure. This is reflected in the incidence of 30-day stroke, pacemaker, and vascular complications requiring endovascular treatment or surgery. In fact, almost one in four patients had a complication event at 30 days post TAVI procedure. This is consistent with previous reports and contributed to the conservative post-procedural approach, including prolonged hospitalisation in this group. However, our data indicate that 22% of urgent TAVI patients in our cohort were able to recover rapidly and were safely discharged on the following day without compromising short-term outcomes.

Data from the TVT registry highlighted comparable pacemaker, stroke and vascular complications among patients who underwent elective versus urgent TAVI [3]. The reported complication rate appears comparable to other studies reporting outcomes of urgent TAVI, including data from our centre [18,19,20]. In contrast, our mortality rate was relatively low as in-hospital mortality was excluded from analysis.

Our study did not include a pre-specified protocol to allow early discharge. The decision was made by the clinical team once the patient was deemed safe and suitable for hospital discharge. This included a few key elements, including satisfactory vascular haemostasis and stable rhythm without a high-degree AV block requiring intervention. Given that patients have been admitted with unstable symptoms, it was essential to ensure that they were safe to mobilise and to return to their baseline function so they can be safely discharged from hospital. The absence of excess adverse events at 30 days in the early discharge group further supports the notion that next-day discharge can be considered in selected urgent TAVI patients.

The observed differences between discharge groups should be interpreted in the context of confounding by indication and post-treatment selection. Discharge timing was not randomised or prospectively assigned but was determined after assessment of clinical recovery, including the occurrence of procedural complications. Patients with uncomplicated recovery were therefore inherently more likely to undergo next-day discharge, whereas patients experiencing complications were more likely to remain hospitalised. Consequently, the lower observed complication rate in the next-day discharge group should not be interpreted as evidence that earlier discharge itself reduces complications or is superior to prolonged hospitalisation. Rather, our findings support the feasibility of next-day discharge in appropriately selected patients following satisfactory post-procedural recovery.

Beyond vascular complications and the STS score, we did not identify any clinical variable that contributed to extended in-hospital length of stay. This included the use of the balloon-expandable valve as previous data suggested that it was associated with better 30-day outcomes for urgent patients undergoing TAVI procedure [3]. The STS score was identified as an independent predictor of prolonged in-hospital stay. However, this correlation was only modest and was associated with a less-than-one-day increase for the total in-hospital duration. Nonetheless, it underscores the relevance of global surgical risk assessment even in urgent TAVI settings. Higher STS scores likely reflect increased frailty, greater comorbidity burden, and reduced physiological reserve, all of which may necessitate extended monitoring. Notably, age, sex, valve type, and pre-procedural haemodynamic parameters were not associated with the length of stay, suggesting that anatomical and procedural factors may be less influential than overall clinical risk.

Developing any complications did not translate into extended in-hospital length of stay. In fact, more than 10% of patients who had short in-hospital stays reported complications following their TAVI procedures. This included major events such as stroke or vascular complications requiring endovascular treatment. Moreover, stroke and pacemaker were not identified as independent predictors of prolonged in-hospital stay. A recent study evaluated the impact of same-day pacemaker insertion in patients developing a high-degree AV block during TAVI procedures [21]. In this multi-centre study of 584 patients, almost one-third of patients underwent same-day pacemaker insertion highlighting the feasibility of this approach without increased risk of pocket haematoma [21]. Importantly, this group was associated with 3 days of reduction in the total length of stay compared to patients who were observed for potential conduction recovery [21]. On the other hand, vascular complication was associated with a 3-to-4-day increase in the total in-hospital length of stay. The association between vascular complications, bleeding and requirement for blood transfusion may explain this finding [22,23,24]. A recent study reported an extended in-hospital duration of more than 7 days in patients who developed vascular complications [22].

Our findings have important implications for clinical practice and resource utilisation. As TAVI volumes continue to rise globally, optimising hospital efficiency without compromising patient safety is essential. Early discharge strategies in urgent TAVI contribute to improved bed availability, reduced healthcare costs, and enhanced patient experience. Nevertheless, careful patient selection remains essential, and early discharge should be reserved for individuals meeting clear clinical stability criteria.

### 4.1. Study Strengths

This study included a consecutive, real-world cohort of patients undergoing urgent TAVI, a population that has largely been excluded from previous studies of early discharge pathways. Data were collected prospectively within a dedicated and routinely audited institutional database, with systematic assessment of clinical, procedural, echocardiographic, and 30-day outcome data. The study also provides practical information on the post-procedural factors associated with hospital length of stay, which may inform the development of structured discharge pathways for selected patients undergoing urgent TAVI.

### 4.2. Study Limitations

This study has several limitations. First, this was a single-centre observational study, which limits generalisability and precludes causal inference regarding the comparative safety of next-day versus prolonged hospitalisation. Second, discharge timing was not randomised or prospectively assigned but was determined following the assessment of post-procedural clinical recovery. Patients with uncomplicated recovery were therefore inherently more likely to undergo next-day discharge, resulting in confounding by indication and post-treatment selection bias. Accordingly, the observed lower complication rate among patients discharged the following day should not be interpreted as an effect of the discharge strategy itself. Third, the primary discharge-stratified analysis was conditional on survival to the point at which discharge could be considered; exclusion of procedural and pre-discharge deaths therefore introduces survivor-selection. To address this, overall 30-day mortality was additionally evaluated in the complete consecutive urgent-TAVI cohort including procedural and in-hospital deaths. Fourth, no prospectively mandated numerical discharge algorithm was used, and discharge decisions remained individualised according to treating-team assessment. Finally, 30-day readmission data were not systematically captured, particularly because patients were referred from multiple hospitals and subsequent admissions could occur outside our institution. Readmission could therefore not be reliably compared between discharge groups.

### 4.3. Future Perspectives

Prospective multi-centre studies are needed to validate these findings in broader urgent TAVI populations and to develop standardised discharge criteria incorporating haemodynamic stability, conduction status, vascular access assessment, renal function, haemoglobin, mobility, and social support. Future studies should also evaluate readmission, patient-reported outcomes, and healthcare costs, and should compare protocol-driven early discharge with usual care using methods that account for treatment selection bias. Such studies should ideally prospectively define discharge eligibility before post-procedural outcomes are known, thereby reducing selection bias inherent in retrospective comparisons based on actual discharge timing.

## 5. Conclusions

Next-day discharge appears feasible in carefully selected patients undergoing urgent TAVI and was not associated with excess 30-day mortality in this observational cohort. These findings should be interpreted in the context of post-procedural patient selection and survivor bias and do not establish the comparative safety or superiority of an early discharge strategy. Prospective multi-centre studies using standardised discharge criteria are required to validate these findings.

## Figures and Tables

**Table 1:** Baseline clinical characteristics of included patients, stratified according to their in-hospital length of stay.

	Whole Cohort n = 279	Short Stay n = 62	Long Stay n = 217	*p* Value
Age _(mean ± SD)_	81 ± 7	82 ± 9	81 ± 7	0.34
Male gender _(*n*, %)_	155 (56%)	35 (57%)	120 (55%)	0.87
Body mass index _(mean ± SD)_	28 ± 6	27 ± 6	28 ± 6	0.12
Diabetes _(*n*, %)_	92 (34%)	15 (24%)	77 (36%)	0.075
Hypertension _(*n*, %)_	182 (66%)	38 (61%)	144 (68%)	0.36
Obstructive lung disease _(*n*, %)_	54 (20%)	17 (27%)	37 (17%)	0.077
Creatinine _(mean ± SD)_	113 ± 64	112 ± 67	113 ± 64	0.92
Haemoglobin _(mean ± SD)_	121 ± 20	122 ± 20	120 ± 20	0.49
Coronary artery disease _(*n*, %)_	92 (34%)	19 (32%)	73 (34%)	0.74
Peripheral vascular disease _(*n*, %)_	23 (8%)	7 (11%)	16 (7%)	0.33
Previous CVA/TIA _(*n*, %)_	27 (10%)	8 (13%)	19 (9%)	0.33
Previous CABG _(*n*, %)_	10 (4%)	2 (3.4%)	8 (4%)	0.89
Atrial fibrillation _(*n*, %)_	91 (33%)	26 (43%)	65 (30%)	0.062
LBBB _(*n*, %)_	47 (17%)	7 (11%)	40 (19%)	0.17
RBBB _(*n*, %)_	38 (14%)	7 (11%)	31 (15%)	0.52
Pacemaker in situ _(*n*, %)_	19 (7%)	1 (2%)	18 (8%)	0.067
STS score _(mean ± SD)_	5.6 ± 3.2	5.6 ± 3.2	5.6 ± 3.2	0.91
Valve-in-valve _(*n*, %)_	17 (6%)	4 (7%)	13 (6%)	0.88
balloon-expandable _(*n*, %)_	110 (39%)	25 (40%)	85 (39%)	0.87
Valve size _(median, IQR)_	26 (26–29)	29 (26–29)	26 (26–29)	0.13

CABG: Coronary artery bypass graft; CVA: Cerebrovascular event; LBBB: Left bundle branch block, RBBB: Right bundle branch block; SD: Standard deviation; TIA: Transient ischemia attack; TAVI: Transcatheter aortic valve implantation.

**Table 2:** Echo characteristics of included patients, pre- and post-procedural, stratified according to their in-hospital length of stay.

	Whole Cohort n = 279	Short Stay n = 62	Long Stay n = 217	*p* Value
Maximum gradient pre-TAVI _(mean ± SD)_	76 ± 26	72 ± 22	77 ± 27	0.25
Mean gradient pre-TAVI _(mean ± SD)_	47 ± 18	45 ± 15	48 ± 18	0.32
Aortic valve area pre-TAVI _(mean ± SD)_	0.71 ± 0.21	0.71 ± 0.21	0.71 ± 0.20	0.97
Left ventricle function pre-TAVI _(mean ± SD)_	47 ± 13	49 ± 12	46 ± 13	0.16
Mitral regurgitation (≥moderate) _(*n*, %)_	46 (19%)	12 (21%)	34 (18%)	0.64
Tricuspid regurgitation (≥moderate) _(*n*, %)_	23 (10%)	6 (11%)	17 (9%)	0.76
Maximum gradient post-TAVI _(mean ± S D)_	16 ± 8	17 ± 7	16 ± 9	0.65
Mean gradient post-TAVI _(mean ± SD)_	9 ± 5	9 ± 4	9 ± 5	0.85
Left ventricle function post-TAVI _(mean ± SD)_	49 ± 11	50 ± 11	49 ± 11	0.47
Aortic regurgitation (≥moderate) _(*n*, %)_	8 (3%)	2 (4%)	6 (3%)	0.80
Mitral regurgitation (≥moderate) _(*n*, %)_	30 (12%)	8 (15%)	22 (12%)	0.44
Tricuspid regurgitation (≥moderate) _(*n*, %)_	24 (10%)	4 (8%)	20 (11%)	0.55

SD: Standard deviation; TAVI: Transcatheter aortic valve implantation.

**Table 3:** Clinical outcomes of included patients, stratified according to their in-hospital length of stay.

	Whole Cohort n = 279	Short Stay n = 62	Long Stay n = 217	*p* Value
Death at 30 days	1 (0.4%)	1 (1.6%)	0 (0%)	0.22
Any complications at 30 days	71 (25%)	7 (11%)	64 (30%)	0.004
Major complications	33 (12%)	3 (5%)	30 (14%)	0.053
Stroke	13 (5%)	1 (2%)	12 (6%)	0.19
Disabling stroke	3 (1%)	0 (0%)	3 (1%)	0.47
Permanent pacemaker	35 (13%)	4 (7%)	31 (14%)	0.10
Vascular complications requiring endovascular or surgical repair	11 (4%)	1 (2%)	10 (5%)	0.29

**Table 4:** Predictors of in-hospital length of stay.

	Model 1 Stroke		Model 2 Vascular		Model 3 Pacemaker	
	B Coefficient (95% CI)	*p* Value	B Coefficient (95% CI)	*p* Value	B Coefficient (95% CI)	*p* Value
Age	−0.08 (−0.19, 0.04)	0.18	−0.08 (−0.19, 0.03)	0.14	−0.08 (−0.19, 0.03)	0.17
Gender	−0.19 (−1.73, 1.35)	0.81	−0.13 (−1.64, 1.39)	0.87	−0.35 (−1.90, 1.19)	0.65
STS score	0.37 (0.11, 0.64)	0.006	0.38 (0.12, 0.64)	0.004	0.37 (0.10, 0.63)	0.007
LV function	0.01 (−0.06, 0.07)	0.87	0.01 (−0.05, 0.07)	0.76	0.04 (−0.06, 0.06)	0.89
Aortic valve mean gradient	−0.01 (−0.05, 0.04)	0.79	−0.01 (−0.05, 0.04)	0.78	−0.01 (−0.06, 0.03)	0.60
Balloon-expandable valve	−0.61 (−2.08, 0.87)	0.42	−0.52 (−1.98, 0.93)	0.48	−0.50 (−1.97, 0.97)	0.51
Complications	1.57 (−1.65, 4.79)	0.34	3.41 (0.98, 5.85)	0.006	1.69 (−0.45, 3.82)	0.12

## Data Availability

The anonymised data supporting the findings of this study are available from the corresponding author upon reasonable request, subject to institutional approval and applicable data protection requirements. The data are not publicly available because they contain confidential patient-level clinical information.
